# Mars and the ESA Science Programme - the case for Mars polar science

**DOI:** 10.1007/s10686-021-09760-6

**Published:** 2021-06-09

**Authors:** Nicolas Thomas, P. Becerra, I. B. Smith

**Affiliations:** 1 Physikalisches Institut, University of Bern, Sidlerstr. 5, CH-3012 Bern, Switzerland; 2 Earth and Space Science and Engineering, Lassonde School of Engineering, York University, Toronto, Canada

**Keywords:** Mars, Mission, Polar caps, Dynamic processes

## Abstract

Current plans within the European Space Agency (ESA) for the future investigation of Mars (after the ExoMars programme) are centred around participation in the Mars Sample Return (MSR) programme led by NASA. This programme is housed within the Human and Robotic Exploration (HRE) Directorate of ESA. This White Paper, in response to the Voyage 2050 call, focuses on the important scientific objectives for the investigation of Mars outside the present HRE planning. The achievement of these objectives by Science Directorate missions is entirely consistent with ESA’s Science Programme. We illustrate this with a theme centred around the study of the Martian polar caps and the investigation of recent (Amazonian) climate change produced by known oscillations in Mars’ orbital parameters. Deciphering the record of climate contained within the polar caps would allow us to learn about the climatic evolution of another planet over the past few to hundreds of millions of years, and also addresses the more general goal of investigating volatile-related dynamic processes in the Solar System.

## Background

Currently, the study of Mars is strongly oriented towards the search for past life and the potential for human exploration. Within the European Space Agency (ESA), this has led to most of the activity connected to Mars research being within the Human and Robotic Exploration (HRE) Directorate of ESA. In this paper, submitted to ESA in response to the Voyage 2050 Call for White Papers, we wish to emphasize that the Science Programme has a significant role to play in the investigation of Mars, and that goals distinctly separate from those of the HRE Directorate, can be formulated. We show this by presenting a case for a Mars Polar Science theme within Voyage 2050.

The Science Programme of ESA was instrumental in placing Europe into the global Mars community through Mars Express. This mission recovered science originally initiated by European investigators as part of the Russian Mars ‘96 mission, and resulted in a strong European presence in the international community that has continued until today. This has led to active instrument provision and participation in missions, the most recent example being NASA’s InSight. The European experiments on InSight had their genesis in Science Programme studies of InterMarsnet (including studies before this of NetLander; [32, 33]), which ultimately lost out to Planck in the ESA Science Programme selection process. European participation in NASA missions such as Mars Pathfinder, Mars Polar Lander, Phoenix, and Curiosity further indicate the scientific enthusiasm for studying Mars through landed static and mobile missions.

The initiation of the ExoMars programme, through studies carried out in the late 1990s, began a change in direction within ESA. ExoMars is part of the optional programme and is housed within the HRE Directorate. The ExoMars programme comprises two missions: (1) a surface platform and rover, which is intended to launch in 2022, and which will attempt to drill into the sub-surface of Mars to test for extinct and/or extant life - it is the most comprehensive astrobiology mission to be launched by any agency so far; and (2) the Trace Gas Orbiter (TGO) which was launched in 2016. TGO was primarily foreseen as a communications orbiter for landed assets (including the ExoMars rover) but available mass was subsequently used to carry scientific payload. This included the Colour and Stereo Surface Imaging System (CaSSIS) instrument led by Switzerland, and the NOMAD spectrometer led by Belgium. The Science Programme contributes to TGO by providing small but significant levels of funding to support science operations. Together with the continued operation of Mars Express, this is currently the only contribution of the Science Programme to the scientific exploration of Mars and, at the time of writing, there are no plans to expand this, to our knowledge.

The situation in Europe is thus one in which HRE is now leading an optional Mars programme, and should ExoMars be successful, ESA (through HRE) will seek to participate in Mars Sample Return (MSR) as a joint international mission with NASA (and possibly other agencies). This mission could be a precursor to the human exploration of Mars, which would be consistent with the perceived aims of the HRE Directorate. However, there are numerous targets and investigative goals at Mars that are of major scientific significance but that are not easily coupled to MSR and are unlikely to be a goal of the HRE programme.

In the following, we will provide an example of such targets and goals: the study of the Martian Polar Ice Caps. Our aim here is to voice the interest of the community in furthering studies of the Martian polar regions, to propose that the Science Programme remain open to these ideas, and to ensure that competitive, Mars-related, science mission proposals are welcome. In addition, though missions to Mars are currently excluded from the New Frontiers-class Announcements of Opportunity following statements in the previous Decadal Survey, the latest goals of the Mars Exploration Program and Analysis Group suggest that this could change in the near future (MEPAG 2020). Should this occur, an ESA Science Programme participation in a Mars-related New Frontiers mission could be scientifically very valuable.

## Scientific justification for a Mars polar science theme within the ESA Science Programme

**Fig. 1:**
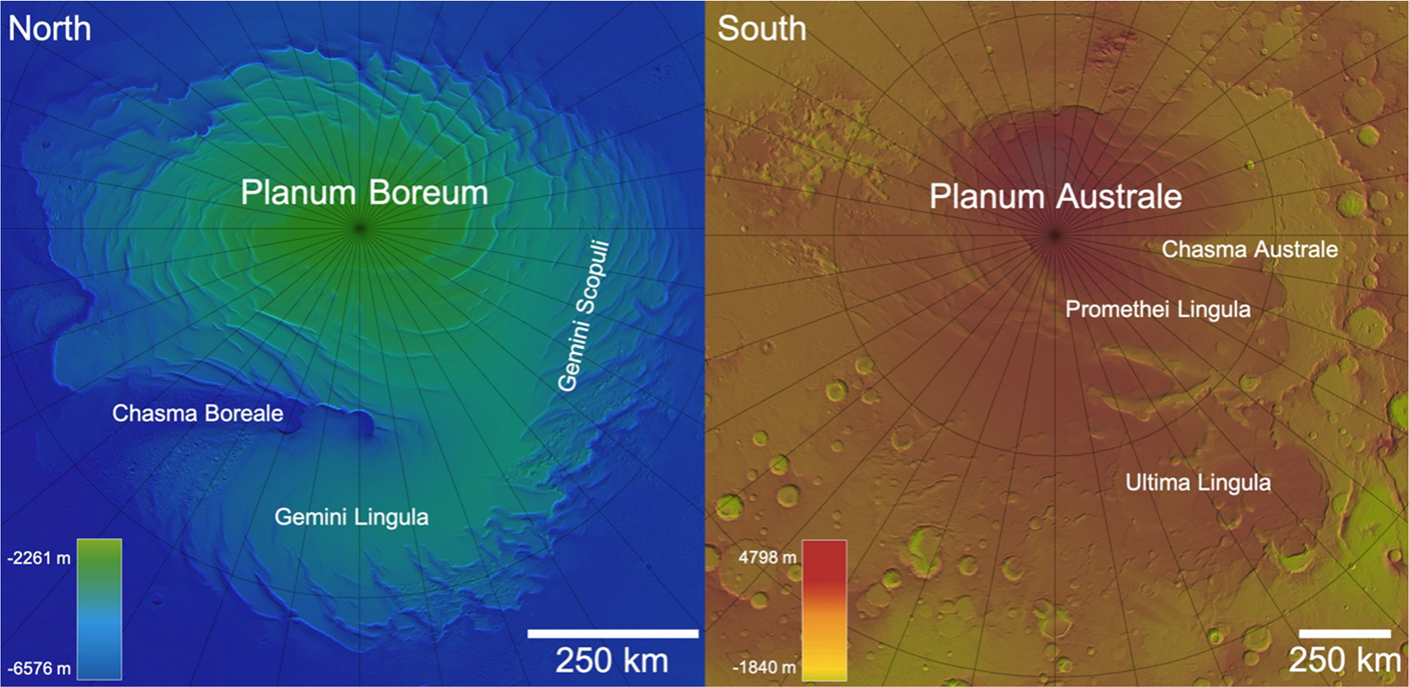
Blended HRSC-MOLA topographic maps of the polar regions of Mars with a few important geographic locations indicated. Latitude and longitude lines are drawn every 10°. (Credit: MOLA data – NASA, HRSC data – ESA/DLR/FU Berlin)

In a broad sense, the interaction of Mars’ polar regions (Fig. 1) with the Martian planetary climate system can be split into three distinct timescales. The seasonal polar caps are produced by the annual mass transfer of CO_2_ between the atmosphere and the surface. These caps exist only during winter and comprise a 1–2 m thick layer of mostly CO_2_ ice. The polar residual caps that remain in summer months are composed of H_2_O ice deposits in the north (the North Polar Residual Cap - NPRC) and CO_2_ ice in the south (SPRC), and they interact with the current Martian climate on timescales of decades to 100 s of years [11], growing to at most a few metres in the north and up to ten meters thick in the south. Finally, the Polar Layered Deposits (PLD) are kilometres-thick stratified sheets of nearly pure water ice [19, 37, 39, 54], with small amounts of dust, trapped gases, and other refractory material, and they record climate oscillations, in an analogous way to terrestrial polar ice sheets and Milankovitch Cycles, from the last few to hundreds of millions of years of Martian history.

The polar environment therefore comprises geological information on the current state of Martian climate, as well as its relatively recent history. Deep understanding of the connection between the polar deposits and the Martian climate is necessary to understand the Martian climate system as a whole, and is only possible through missions that are dedicated to studying this record in detail.

### The seasonal deposits

Mars’ seasonal polar caps buffer the CO_2_ atmosphere of Mars [31]. Surface pressure changes of around 25% occur over the annual cycle as a result of mass transfer between the polar caps and the atmosphere with CO_2_ freezing out onto the polar caps in winter and subliming in spring and summer.

**Fig. 2:**
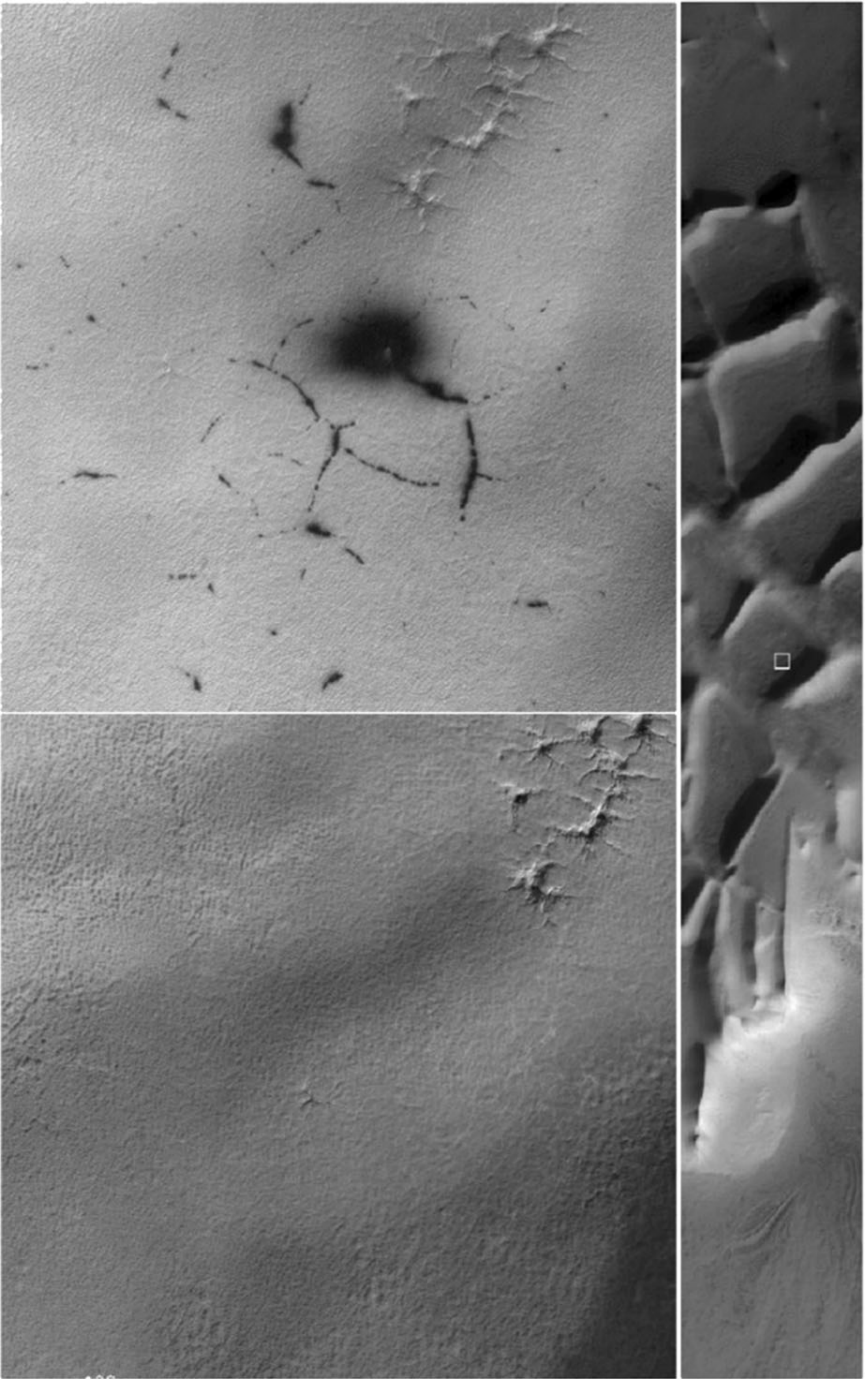
Evidence of gas jet activity in the “Inca City” region of the southern polar cap (~81°S). The right-hand panel shows the full image of the area (taken by NASA’s High Resolution Imaging Science Experiment (HiRISE)). A white box identifies the area shown in more detail in the left two panels. These two images were taken at different times. Black blotches and lineaments appear in spring as a result of the Kieffer mechanism for producing CO_2_ gas geysers. (From [51]. (Credit: NASA/U Arizona/HiRISE))

This leads to numerous small-scale processes that are active and dynamic on seasonal timescales. Figure 2 shows an example of the types of activity commonly seen in the southern hemisphere [51]. During southern winter, CO_2_ condenses slowly onto the southern polar cap and produces a roughly 1 m thick layer of translucent CO_2_ ice. As the Sun rises in southern Martian spring, sunlight penetrates the layer and illuminates the surface below. The CO_2_ layer is opaque at infrared wavelengths, inhibiting heat escape, which causes sublimation of the CO_2_ layer from its base, resulting in gas pressures that force openings and cracks in the CO_2_ layer and release CO gas in a geyser-like process. Pressure release is accompanied by dust transport leading to dark deposits on the surface of the ice (Fig. 2 top left). This mechanism scours the surface beneath the ice layer and produces so-called araneiform (spider-like) surface structures (as seen in the top right of the high-resolution images). This mechanism was first described by Kieffer et al. [26], and modelling work has placed constraints on the physics of the gas emission (e.g. [52]). However, we have never seen these processes “in action” despite considerable efforts to do so. This may be because the dust in the jets is optically too thin, and what we see is the result of continuous outgassing with low dust content over periods of several hours or even days. The whole process may be analogous to that seen at Triton where the volatile involved is probably nitrogen. On Triton, however, we have seen active geysers indicating a higher non-volatile content and allowing a better assessment of their properties [50].

**Fig. 3:**
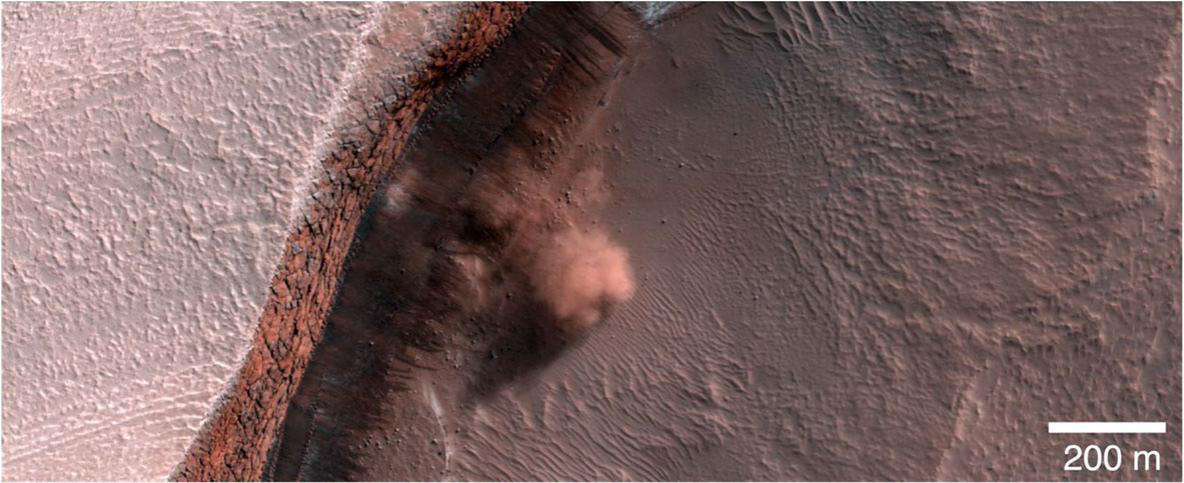
HiRISE image ESP_016423_2640 showing an avalanche from a scarp that cuts into the margins of the topographical dome of Planum Boreum in the north polar region of Mars (~83° N). (Credit: NASA/U Arizona/HiRISE)

The CO_2_ burden at specific sites on the surface leads to other phenomena. Pilorget and Forget [40] have described how gully-formation in polar regions may be triggered by the CO_2_ sublimation and condensation cycle. On the margins of the Planum Boreum dome of the North polar cap, massive dust-ice avalanches could possibly be triggered by CO_2_ ice sublimation ([43] and Fig. 3) although more recent work suggests that the avalanches are triggered by thermal expansion of water ice when heated in spring [4, 13]. These avalanche events are extremely numerous and observations by NASA’s Mars Reconnaissance Orbiter (MRO)‘s High Resolution Imaging Science Experiment (HiRISE) have frequently caught more than one avalanche within one image swath, which typically take <10 s to acquire. In Fig. 3, the bright surface to the left is the relatively smooth, flat plateau that comprises the surface of the NPRC. The reddish, fractured deposits in the middle of the image are H_2_O ice-rich layers exposed at the steep margin of the north polar layered deposits (NPLD) which exhibit varying dust content with depth. At this location, the icy layers exposed along this scarp can receive direct sunlight during summer, and therefore experience intense thermoelastic stresses that may lead to avalanches initiating at various heights on the scarp [4, 13]. Failure and collapse of parts of many scarps along the NPLD margins have been observed over annual timescales by HiRISE. Figure 3 shows the base of the scarp with a cloud of material from one such avalanche captured at the moment of imaging. The residue at the base of the scarp is material that has only recently been exposed. Studying this debris provides access to the sub-surface material of the lowermost exposed layers of the NPLD. Studies of the sublimation of the avalanche debris [16] indicate that the original material is ice-rich. However, it is often difficult to tell where exactly along the scarp the material came from, since a detailed composition-based stratigraphic column at the visible bed-scale is still elusive.

Southern and northern winters are not identical because of the elliptical orbit of Mars, and it is not necessarily the case that processes operating in one hemisphere will be dominant in the other. Southern winters are longer, allowing more CO_2_ to condense and producing thicker ice layers [22]. At high northern latitudes dynamic activity associated with sublimation of the seasonal polar cap is mostly found on dunes and araneiform feature formation is almost certainly completely absent. Instead, activity in the southern polar region occurs mostly on more consolidated substrates and spider-formation is common. The differences are such that it is unclear what changes to the simple application of the Kieffer mechanism are necessary to explain the observed phenomena.

The mass of material condensing on the caps each Mars year has been estimated by gravity field measurements. In combination with altimetry data from the Mars Global Surveyor Spacecraft the depth of the precipitation has been measured to be up to 2 m at each pole. However, the density is suspected to be significantly greater in the south than the north indicating a different form of precipitation in the south, probably direct condensation rather than snow or frost [47].

The evolution of the seasonal caps from year to year allows knowledge of annual variations in the Martian atmosphere. In addition, Planet Encircling Dust Events (PEDE) are a frequent occurrence on Mars, although a reproducible pattern to their occurrence has not yet been established. Nevertheless, more localised storms occur at predictable seasons within the Martian year. The dust elevated during storms, and from much smaller local processes, is transported by atmospheric circulation, and some of it deposits onto the polar caps. Upon sublimation in spring, the deposited dust is left behind as a lag that can be further modified by surface winds. The influence and dynamics of dust transport on Mars and its effects on the polar regions and the Martian environment as a whole is still the subject of active research, given that understanding these effects and their variability is a key aspect in understanding Mars’ climate evolution.

The principal questions that arise from our current knowledge of seasonal processes are

- What is the nature of the activity associated with sublimation of the seasonal polar caps?
- Is the density of the CO_2_ deposited on the polar caps different between the northern and the southern hemisphere and, if so, why?
- Do our current models, built on the Kieffer hypothesis, correctly explain the behaviour of the surface activity in southern spring?
- What are the current deposition rates of H_2_O and dust onto the polar caps as a result of seasonal processes?

### The residual polar caps

**Fig. 4:**
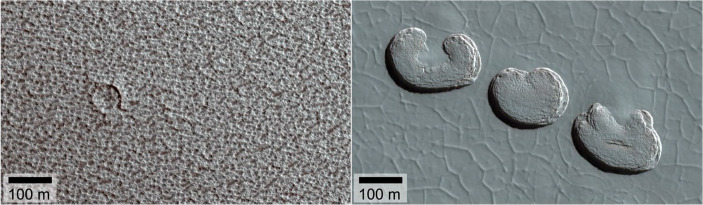
Left: False colour HiRISE image (PSP_001922_2680) of the North Polar Residual Cap showing its pitted texture and a rare impact crater. Right: Sublimation pits of the so-called ‘Swiss cheese’ terrain of the South Polar Residual Cap imaged by HiRISE (PSP_003738_0930). The Sun illuminates the scene from the lower left. Each feature is about 200 m in diameter. (Credit: NASA/U Arizona/HiRISE)

The residual caps, as their name suggests, are composed of young ice that survives the summertime sublimation in each hemisphere. The North Polar Residual Cap (NPRC), is composed of water ice, and is thought to be accumulating actively today [9, 29]. Its surface contains patterns and linear textures that appear to be the result of differential sublimation (Fig. 4, left; [44]). Recent modelling studies suggest that these patterns reflect the conditions of accumulation of the ice of the NPRC [53], which is likely to be the top layer of the NPLD. Thus, understanding the NPRC accumulation provides a baseline with which to understand the process of accumulation and ablation of each NPLD layer. However, understanding the properties of the ice by in situ investigation may be crucial to achieving this task.

The South Polar Residual Cap (SPRC, Fig. 1) is composed primarily of CO_2_ ice over a thin substrate of water ice, and is offset from the geographic pole. According to most general circulation models it is highly unstable [11]. It has a unique geomorphology that provides clues as to how it is even able to survive in the first place. Some of this unusual geomorphology is known as “Swiss cheese” terrain (Fig. 4, right). This name describes areas of the SPRC surface covered with pits that are typically a few hundred metres across and 8–10 m deep. They have flat floors and steep sides. The individual pits grow in diameter through sublimation at an average rate of 1–3 m per year, suggesting that they are formed in a thin layer of CO_2_ ice lying on top of the residual polar cap. These features are therefore modern and transient, reflecting the current orbital configuration of the Martian system [12].

Underneath the SPRC, older deposits of CO_2_ ice were discovered by MRO’s Italian-led Shallow Radar experiment (SHARAD). These reservoirs contain enough CO_2_ to more than double Mars’ current atmospheric pressure [7, 38]. These units are interspaced with thinner bounding layers of water ice, and models of their combined evolution suggest ages of a few hundred thousand years and an intricate interaction with the Martian atmosphere over that timespan [10, 34].

The residual caps also raise important questions directly related to the Martian climate system, namely

- Why is there a difference in composition between the two residual caps?
- What are the processes involved in the emplacement and removal of CO_2_ ice on the south residual cap?
- What is the climate record expressed in the PRC’s, and can we access it?

### The polar layered deposits

**Fig. 5:**
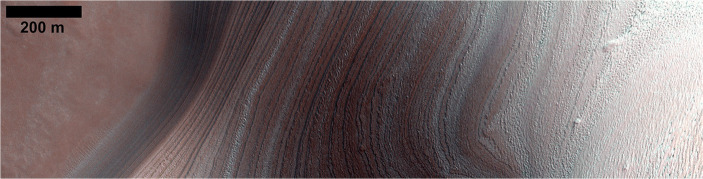
CaSSIS colour image of a bedding exposure along the northernmost margin of the SPLD. (Credit: ESA/Roscosmos/CaSSIS)

The polar layered deposits (NPLD in the north and SPLD in the south) of Mars record signals of climate over millions to hundreds of millions of years of accumulation and modification. Deep troughs and marginal scarps dissect the PLDs in a quasi-spiral pattern and expose layers of ice and dust in a way similar to how the sedimentary record of the Earth is revealed in valleys and canyons. The alternating nature of the PLD structure is believed to be caused by variations in rates of ice and dust accumulation and the product of oscillations in Mars’ orbital parameters. This connection between orbital dynamics and geology is similar to how Milankovitch cycles affect climate, and therefore ice and sediment deposits, on Earth. Observations of the PLD have been obtained by several instruments over the years. An example of a marginal outcrop of the SPLD taken by the CaSSIS imager is shown in Fig. 5.

Variations in the orbital configuration of Mars (obliquity and inclination) dictate the amount of insolation received by different latitudes of its surface, and therefore directly influence planetary climate and the locations where ice can be stable on the surface. The calculated evolution of Mars’ orbital configuration is mathematically stable and robust back to 20 Myr. Before this, the solutions are non-unique and Mars’ orbital motion cannot be constrained [30]. However, over the last 20 Myrs, the obliquity of Mars has varied periodically between 15 and 45 degrees [30], leading to vastly different climates and to ice being periodically re-distributed to various regions of the planet. In general, high values of obliquity mean that the poles receive more sunlight on average than the mid-latitudes, which leads to ablation of polar ice. Conversely, low obliquity promotes accumulation of polar ice [49].

**Fig. 6:**
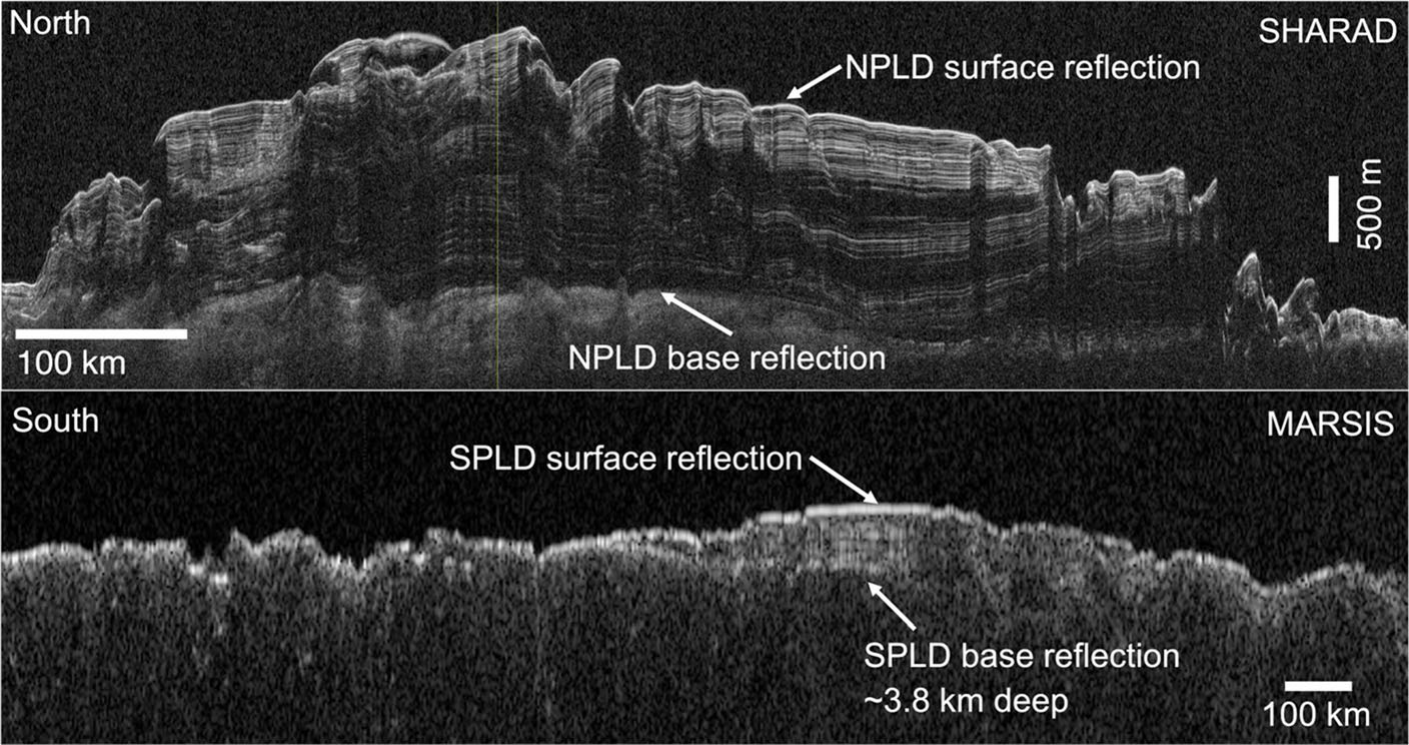
Examples of radargrams from SHARAD and MARSIS showing the sub-surface structure of the NPLD (top) and the SPLD (bottom). (Credit: NASA, ESA, ASI)

Direct investigation of the internal structure of the PLD has also been made by subsurface sounding radar, which showed the uniformity of PLD layers across the entirety of the PLD domes (Fig. 6). The data returned by MARSIS on Mars Express and SHARAD on MRO (both Italian-led experiments) consist of two-dimensional “radargrams” that display the returned power and the time delay between transmission of the radar signal and a subsurface return. Ice is relatively transparent to radar wavelengths, so subsurface returns occur when there is a change in permittivity between a less dusty layer to a more dusty one. Examples are shown in Fig. 6. In the NPLD, the attenuation of the radar signal combined with permittivity models established a maximum bulk dust content of 5% [19]. Similarly, in the SPLD, the dust content was found to be around 10–15% [45]. The relationship between permittivity and dust content is not completely understood [28] although experimental work in this domain is becoming a European strength [8]. The repeated fly-overs resulting from the polar orbits of Mars Express and MRO have led to 3-D maps of the sub-surface that help establish a broad-scale stratigraphy [41, 42, 46].

The observed internal structure allows us to trace layers such as those seen in Fig. 6 through the cap to locations where exposures are absent [35]. However, this has not been fully achieved yet, but research is ongoing [5, 15]. If this correlation is achieved, it could be used to confirm the assumption made by Fishbaugh and Hvidberg [17], Fishbaugh et al. [18], and Becerra et al. [1] that the external appearance and topographic relief of an exposed NPLD layer at one location can be related to a counterpart exposure at a different location. Hence, a stratigraphic column could be produced to obtain a consistent view of the deposition with depth.

Becerra et al. [1–3] have shown that the photometric and topographic expression of layers can be isolated using imaging and digital terrain models of the surface. These data can be studied with time-series (e.g. FFT or wavelet) to attempt to detect signals of the climate variations. Patterns and periodicities in the exposed layering can be compared to the period of the change in obliquity and argument of perihelion precession of Mars. Becerra et al. [2] found a correlation between stratigraphic periodicities in the NPLD and orbital oscillation frequencies. The likely younger age of the NPLD (onset <5 Ma due to a decrease in average obliquity that allowed ice to deposit at the north pole), also allowed them to make estimates of the ages of individual layers, concluding that the top 500 m of stratigraphy accumulated in the last 1 Myrs of Martian history.

The SPLD are older (perhaps 30–100 Myr; [27]), which makes age estimation for individual SPLD beds impossible given the non-uniqueness of the orbital solutions beyond 20 Myr into the past. Nevertheless, Becerra et al. [3] used CaSSIS and HiRISE stereo data to study the SPLD and relate the periodicities in the SPLD stratigraphy to the same orbital frequencies that forced the accumulation of the NPLD. These frequencies should be robust on much larger timescales than the past 20 Myr, even though the specific oscillation solutions are not [30]. It was inferred that the water ice and dust of the SPLD was deposited at variable rates of 0.13–0.39 mm/yr, taking a minimum of 10–30 Myrs to accumulate.

The deposition and ablation of material from the PLD is driven by atmospheric dynamics. The global circulation models used to study the present behaviour of Mars’s atmosphere readily reproduce the CO_2_ cycle (e.g. [20]), as well as the water cycle including cloud physics (e.g. [21]). However, the interannual variability of the dust cycle remains difficult to predict with current models. This shortcoming is important, given that varying dust deposition onto the caps is what allows us to read the signal of climate variation, whether it be in the subsurface radar data or in the images of PLD outcrops. Nonetheless, the predictive capability of Mars Global Circulation Models (GCMs) is improving and this presents the possibility for studying Mars’ atmosphere throughout the Amazonian period by using Laskar et al. [30]‘s orbital parameters. Although GCMs are not fast enough to study dynamical changes in climate, steady-state solutions with past obliquities and inclinations are feasible. The CO_2_ cycle is particularly affected as noted above and seasonal variations become more extreme as the obliquity increases. The regions on the surface where water ice is stable are also changed and current models of dust lifting and loading would predict enhanced dust activity as the obliquity increases [25]. On the other hand, there have been relatively few studies directly related to the PLD – the modelling of Newman et al. [36] is an example although this is now becoming somewhat outdated. Measurements of current atmospheric properties near the PLD surface would provide additional constraints on models of present day Mars and would allow improved extrapolation to earlier times. We note here that GCMs are a strength of ESA and specifically the French and British communities. Europe is the world leader in studying atmospheric dynamics from metre to global scales. These models rely on validation and refinement from observations (assimilation), which can be performed with data obtained in situ or from orbit.

The foregoing suggests that detailed analyses of the PLD would provide a unique opportunity to increase our understanding of the climate history on Mars and test our climate models on a relatively simple terrestrial planet system. A Keck Institute for Space Studies (KISS) workshop held in 2017 entitled “Unlocking the Climate Record Stored within Mars’ Polar Layered Deposits” (see [49]) resulted in a set of four questions that summarized the key open issues to understanding and interpreting the climate record within the PLD:

- What are present and past fluxes of volatiles, dust, and other materials into and out of the polar regions?
- How do orbital forcing and exchange with other reservoirs, affect those fluxes?
- What chemical and physical processes form and modify layers?
- What is the timespan, completeness, and temporal resolution recorded in the PLD?

These issues include studies of the atmosphere, the surface-atmosphere material exchange, and the sub-surface. The same questions were discussed, and their importance reiterated, during the 7th International Conference on Mars Polar Science and Exploration [6]. Through these meetings, it is clear that there is consensus among the Mars Polar Science community on the significance of these issues, and on the fact that addressing them might require multiple missions. Nevertheless, there is also little doubt among the community that major progress could be made with a single mission, and it is undeniable that answering these questions will provide us with a remarkable result –

**we will understand the astronomically-forced climate evolution on another planet.**

## Possible implementations of Mars polar research missions

While there is a strong case to study the seasonal polar caps and the associated dynamic phenomena, here we use as a case study the investigation of the specific properties of the PLD. This is primarily motivated by our goal to study and understand the climate history of Mars over the past few millions of years, given its importance for Martian evolution and planetary science as a whole. It is also the research area that has attracted the most interest from US-based scientists proposing missions to study the Martian poles, but it should be noted that there is plenty of scope for modifying the main scientific focus to respond to the other interests of the European community if required. The present focus of the US community is illustrated by the recent proposal of the COMPASS mission to NASA’s Discovery programme [14]. This mission was designed to look at the PLD using orbital remote sensing only (see below). However, the forthcoming Decadal Survey in the NASA system may recommend that, as from 2023, New Frontiers Announcements of Opportunity should also be open to Mars missions (the currently running Decadal Survey prohibits Mars missions being proposed to this mechanism and this will remain active through to 2023). In preparation for the Decadal Survey, the Mars Exploration Program Analysis Group (MEPAG) recently concluded a study that included various versions of polar landed missions in the Ice and Climate Evolution Science Analysis Group (ICE-SAG).^1^ Hence, a landed mission to the PLD within the New Frontiers programme is feasible in the 2030–2040 timeframe and may provide an opportunity for ESA to collaborate with NASA on a major scientifically important mission to the Martian poles that studies the climate record on a terrestrial planet.

There are several possible approaches to studying the PLD. They are all technically challenging but all provide opportunities for ESA to advance its technical capability in fields where some previous experience exists. These approaches are likely to have significantly different costs and thus a programme or mission proposal could be adapted to meet a specific cost target. We split the possibilities into five categories:

- deep drilling,
- rovers,
- near-surface flight,
- surface networks, and
- orbital reconnaissance

### Deep drilling

Landing on the PLD with a static, fixed lander and drilling downwards is a feasible approach but is probably the most challenging. However, it could also be the most rewarding, as the possibility of dating an ice core on the surface of Mars would bring Mars Climate Science forward by massive leaps, and place our understanding of Martian climate evolution almost on par with how we study our own planet. This approach could be based on drilling methods and technologies studied for Rosetta and the ExoMars rover. In order to make the science return significant, the goal would be to drill to a significant depth. US developments have been exploring whether mechanical drilling to 100 m depth through ice is achievable. Extraction of a core could allow surface manipulation and investigation with large scale instruments; alternatively, instruments could be lowered into the borehole and observations made from within the individual layer. An alternative, albeit with capability to bring fewer instruments, is thermal drilling [23, 49]. In this way, we would obtain the stratigraphy of the uppermost layers at potentially extremely high resolution (<mm) through microscopic imaging, local infrared spectroscopy and even sampling techniques. Another alternative to coring, could be removing debris from the boring process via some form of suction device and feeding collected ice and dust samples into an evolved gas analyser or some form of balance to find the dust content could be envisaged.

Deep drilling would be the optimum approach for a detailed study of the NPLD and previous studies in preparation for Rosetta/Philae as well as ExoMars are of benefit. There are, however, significant difficulties and technological challenges. Specifically, maintaining the borehole open to great depths is challenging, and testing on ice (e.g. Greenland ice sheet) and rock environments (e.g. rock glaciers) would be almost mandatory for any proposed system. Drilling to great depths and maintenance of the integrity of any core acquired while removing it from the borehole require both power and relatively sophisticated mechanics. Failure tolerance is also likely to be critical.

### Roving on the layer exposures

The experience being gained with the ExoMars rover could be used to study the layers by driving over them. The average slope of the PLD troughs that are observed from orbit is <5° and max slopes reach only 12° over a short baseline. This is easily attainable with current technology, so it is quite probable that a capable rover could traverse them. An ExoMars style rover (including its wheel walking capability) may be able to traverse the layers and sample at discrete points. The rover would carry a drill in order to penetrate to below the weathered exposed surface, however, this drill would not need to penetrate to great depths. It is only required to study material a few centimetres to a metre below the surface cover in order to evade the effects of surface weathering. Removed material from the boreholes could then be brought directly to the interior of the rover for analysis. The experience gained with ExoMars is directly relevant (although it is not expected that ExoMars will encounter compacted ice at Oxia Planum).

The main difficulty of this approach is the speed with which the rover can move and the power source needed to maintain a significant enough study. At first, a movement and sampling cycle for one layer may require 5–7 days. This might result in analysis of perhaps 20–30 layers during a summer season before autumn brings CO_2_ condensation. Keeping the rover warm over the winter periods to improve science return is likely to require some form of radioactive heating element. The rover approach possibly provides the best way to investigate individual layers over a large range of depositional history spanning 10^6^ years.

### Near-surface flight over the layers

The potential for planes, helicopters and, most recently, drones have been discussed as a means of providing high resolution studies of sites that are less easily accessible by rovers. Such systems are also able to cover larger distances far more quickly. At Mars, mission proposals such as Kitty Hawk, a plane to fly through Vallis Marineris, have been discussed on several occasions while the recent selection of the New Frontiers mission to Titan, Dragonfly, and JPL’s Ingenuity Mars helicopter shows that NASA sees big advantages in using this type of technology for investigations of objects with thick atmospheres. ESA does not yet possess this technology but its advantages for covering large areas and steeper slopes on the PLD can be easily imagined. Scanning up and down the PLD with a remote sensing suite to provide ultra-high resolution of the individual layers is attractive for studying short-term variability of the Martian climate. The disadvantage is that physical penetration of the surface is unlikely to be feasible and the mass and power of analytical instruments may be restricted because of the low atmospheric pressure. These aspects would need to be studied.

### A network of surface stations

A network of surface stations is a less ambitious concept but nonetheless scientifically attractive. At its current stage of evolution, we do not understand well the present-day fluxes of volatiles and dust into and out of the polar regions. These fluxes are likely to be position dependent on, for example, the NPLD, where local atmospheric dynamics is affected by strong topographic forcing [24, 48]. The atmospheric dynamics at the poles are also of importance and landed measurements of wind speed and pressure would be highly valuable additions to more equatorial measurements. Hence a network of small landed stations to study the variations in upward and downward fluxes of CO_2_, H_2_O, dust, and minor species over one polar cap is a concept that could be investigated. By attempting to characterize fully the present-day evolution of the polar caps and the local atmospheric dynamics, we can relate the thicknesses of the layers detected by other missions to local weather. The observations would also provide important constraints on global circulation models.

### Studies from orbit

The present fleet of Mars missions includes the SHARAD instrument on MRO. However, instruments with other performance parameters can add significantly to our knowledge. At higher frequencies, the uppermost layers of the PLD can be studied at >10x improved vertical resolution. In a suitable near-polar orbit, a combination of sub-mm sounding and thermal infrared spectroscopy would allow determination of local atmospheric and surface properties (wind speeds, water vapour content, and local surface temperatures) over both caps. This global study would be the simplest and cheapest option (as is shown by the COMPASS proposal fitting in NASA’s Discovery programme; [14]) but information on surface fluxes of various species at the surface will be limited.

## Relationship to a theme

We suggest that a Solar System theme to study current evolutionary processes or dynamical processes would be inclusive and could form an umbrella over scientific goals relevant to many objects within our Solar System. These may include Enceladus, Triton, Io, Venus, the lunar polar caps, comets, and, as we have outlined here with specific reference to the polar caps, Mars. Such a theme could act as a focus for future L- and M-class missions while providing an anchor point for future Missions of Opportunity in response to New Frontiers calls within NASA’s programme. Given the timescale of Voyage 2050, listing possible targets and missions seems more appropriate than actually making a pre-selection at this stage. Here, we have pointed out that the Science Programme has a role to play at Mars and there is a strong case for including Mars-related science goals within Voyage 2050 themes.

## Conclusions

We have argued here that scientifically important goals at Mars are in danger of not being covered by either the Human and Robotic Exploration Directorate or the Science Directorate at ESA. We have illustrated that the Martian polar caps are examples of targets that are vitally important to understanding how the Martian climate has evolved over the past 5 Myr to 100 s of Myrs and the evolution of terrestrial planet climates. They are unlikely subjects of HRE-led missions in the near future. We therefore propose that the Science Programme remains open to these ideas within its programme and ensures that competitive, Mars-related, science mission proposals are welcome – possibly within a Solar System theme centred around current evolutionary or dynamical processes.

The study of the Martian polar regions can provide critical understanding of the climate system of Mars, which can be useful to understand planetary climate change on any terrestrial planet. This is in itself an important research theme in disciplines from Earth Science to Exoplanetary astronomy and geophysics. Furthermore, Mars-related missions may re-enter NASA New Frontiers planning in the near future through the next Decadal Survey (they are currently excluded by the Decadal Survey of 2011) and an ESA Science Programme participation in a Mars-related New Frontiers mission would be scientifically valuable and of major interest to the European Solar System community.

## Data Availability

No data used.

## Footnotes

1 https://mepag.jpl.nasa.gov/reports/ICESAG_Report_FINAL.pdf

## Code availability (software application or custom code)

No code used.
